# Editorial

**Published:** 1864-01

**Authors:** 


### Editorial.

### SMALL-POX HOSPITAL.

As many of our readers may have seen alarming accounts of the prevalence of Small-Pox in this City, in the daily papers, and still more alarming accounts of the condition of the Pest-House or Small-Pox Hospital, we copy the following report of the condition of this institution, made by Dr. BARTLETT to the Chicago Medical Society a short time since. The report is perfectly accurate in its statements, and furnishes a good illustration of the inadequate attention generally paid to the most important Sanitary matters, by the Municipal Authorities of some of our large cities.

There is really no more cause for alarm concerning the prevalence of Small-Pox in the city now than there has been at any time during the last four months. When our Legislative and Municipal Authorities will adopt efficient and compulsory measures for having every child vaccinated before it is two years old, and re-vaccinated between the ages of fifteen and twenty years, Small-Pox will be essentially expelled from the community, but not until then.

Dr. BARTLETT'S report is as follows:—

Mr. PRESIDENT.—The committee appointed at the last meeting of this society, to investigate the condition and management of the City Pest House, report as follows:—

"The Pest House was established in October, 1862. Dr. L. P. Cheney is physician to the institution, and Mr. C. S. Perry is the directing officer. It is situated near North Ave., between Clark Street and the lake. The site is unobjectionable, except that the potter's field lies within twenty rods of it. The building is an old one-story frame, fourteen feet wide and sixty-eight feet long, having a small L at the westerly end. The main part of the house is divided into four rooms, communicating with one another, each fourteen by seventeen feet. The L is divided into two very small and low rooms for the nurses. The westerly room is the kitchen, the three others are wards. Each is lighted by two windows, placed opposite one another in the north and south walls. Two of them have outside doors. These three rooms have an air-space of 6,426 cubic feet. Means of warming the house seemed ample, though no instruments are used to regulate the temperature. The floors gave evidence of recent scrubbing; but in places they were foul with spittle and expectoration. There were no spittoons; the sputa of the bad cases were received in chamber-pots, each of which contained urine. The walls were black and dirty.

The windows were all closed. The outside doors of the second and third rooms stood ajar, and there was an open pane in the window of the first ward. Drafts of air were admitted, which might be deemed to be as unsafe to some of the patients as they were salutary to others. No proper means for permanent ventilation exist. The atmosphere of the rooms was not, at that time, more offensive than a badly regulated hospital for ordinary diseases. The peculiar odor of small-pox was not perceptible, except in the immediate vicinity of the worse cases. No disinfecting agents are employed.

In the female ward there were six beds; in the second room, seven; and in the third, six. For bedsteads, army-cots are used; upon these single mattresses are placed. The bedclothes consist of cotton quilts only. Some of the patients lay on mattresses, nearly all had one or more comforters under them. There were, with one or two exceptions, no sheets or pillow-cases used; though the Health Officer stated that a quantity of these articles were on hand. The beds and bedding appeared very filthy, and there is reason to believe they were so. The quilts are aired, but never washed. After bad cases, the mattresses are burned; commonly, they are filled with new straw. Several badly soiled cots and mattresses were observed in the yard, which, it is presumed, were to be used after airing.

There is but little hospital clothing for patients.

There are no screens for the dying. There were in all twenty-five patients—eighteen males, five females, and two children. Four were dangerously sick, nine were progressing favorably, and twelve were convalescent. The cleanliness of the patients seemed to be entirely neglected. The medical attendanr stated that, not so much as their faces were regularly washed, and this was done only when they were uncommonly filthy from effusions of blood or matter. Your committee saw much to confirm this statement. Sometimes the physician has had occasion to reprove the nurses for delay or neglect in removing the excretions of bad cases, but generally there has been no ground for complaint. One case of serious delinquency on the part of the dresser fell under observation.

The convalescents are allowed the liberty of the house. The second ward has an air-space of about 2,142 cubic feet. This is less than the minimum space set apart in well-regulated hospitals for *two* patients. Beside the seven patients belonging in this room, there were, at the time of the visit, four convalescents. Six of these men were negroes.

The clothes of persons entering the hospital are put out to air. No patient is allowed to go out without a written discharge from the physician.

No opportunity was had to ascertain the diet of the patients. There is no diet table.

The kitchen was perfectly neat, and the larder not wanting in good, substantial articles of food. It was stated that any diet could be procured commonly used in hospitals.

Two women and one man, with the assistance of convalescents, discharge all the duties of the house. The physician visits the hospital as occasion requires, usually once daily. The treatment employed by him does not materially differ from that generally adopted. In mild cases he pursues the expectant course. He allays undue excitement in the beginning with antimonials. When the secondary fever assumes a low form, or if exhaustion from any other cause threatens, strength is sustained by strong broths and quinine. Occasionally alcoholic stimulants are necessary, but they are not thought so generally applicable as preparations of bark. Dr. Cheeney attaches great importance to securing sleep. With him, opium is a favorite medicine throughout the disease.

The sarracenia purpurea has not been tried. The sulphite of soda has been used with some apparent advantage.

Of late, no applications have been made to prevent pitting. Dr. Cheney has not found those means, the use of which seemed practicable under the circumstances, to be of much benefit. He stated, however, his intention to put in use other new methods from which he anticipated success. The rooms are kept darkened and, as a rule, there is no pitting.

The mortality has been very slight. Since the establishment of the hospital, 199 have been treated. Of these 36 died—a death-rate of a little over eighteen per cent. Dr. Cheney may very well congratulate himself upon so favorable a record, especially when the disadvantages under which he has labored are duly considered.

On the day of the visit of your committee, a new ward was being opened. It forms an L with the east end of the main building. It is 17 by 18 feet, with a height of ceiling of 7 feet 6 inches. Two small half windows on either side will be the only means of ventilation.

In conclusion, your committee would state that every facility for obtaining the desired information has been courteously afforded them by the physician in attendance. The Health Officer also gave his assistance.

On motion the report was adopted.

---

### UNITED STATES SANITARY COMMISSION.

*To His Excellency the President of the United States:*

Sir:—The United States Sanitary Commission, authorized by the Government to act as a Commission of Inquiry and Advice in respect to the sanitary interests of the national forces, have been for more than two years and a half close and careful students of the medical and hygienic affairs of the army. They ought to be, they are thought by the people of the United States to be, they claim to be, better acquainted with the working of the Medical Department, whose deficiencies, mistakes, and necessities, it is their solemn duty to discover and obviate, than any other responsible body of witnesses. Trusting in their discretion, zeal, and works, the people of the loyal States have made them their almoners, to the extent of seven millions of dollars' worth of sanitary stores, and a million of dollars in money. The disbursement of this immense charity has brought our agents into close and continual contact with the Medical Department, to whose steady and rapid improvement from the imperfect state in which we found it, to its present degree of surprising and gratifying efficiency, we are able to lend a most indisputable testimony. We attribute this immense improvement to the fact that for two years the Medical Department has been directed by Dr. W. A. Hammond, Surgeon-General, a man known to all impartial and competent judges, as thoroughly scientific, highly endowed, large-minded, and an energetic and controlling administrator. He was selected for his office solely for fitness, and in our calm and deliberate judgment, his administration has more than justified all the high hopes and expectations of those who recommended him for the place.

We hear from sources that do not permit us to doubt the fact, that cautious but systematic efforts are now making to remove Surgeon-General Hammond from office. In the name of some millions of constituents, in the name of the homes of this country, whose solicitude, liberality, and watchfulness we represent, we respectfully and conscientiously protest against the secret tribunal, and the indirect methods by which the good fame of the Surgeon-General has been already seriously, and we believe unjustly, aspersed. We protest in our character of experts, a body whose business it has been made to inquire and advise on this very subject, that the removal of Dr. Hammond would be as serious a blow at the lives, comfort, and efficiency of the army as the enemy itself could inflict; that the science of the country, the humanity of its homes, and the army itself, would resent it as a cruel wrong and an alarming error; and we feel ourselves bound, in the interests of the soldiers in the field, and of those about to enter the service of the country, in defense of our own principles and convictions, and in the name of the science, the charity, and the fair-mindedness of the nation, to beg that no further steps in this direction may be taken, without a full and fair trial of Surgeon-General Hammond upon the charges alleged to have been secretly made against him.

*December* 29, 1863.

Signed {

H. W. Bellows,

Wm. H. Van Buren,

Wolcott Gibbs,

Geo. T. Strong,

C. R. Agnew,

}

Standing Committee U. S. Sanitary Commission.

We copy the above very singular document from one of our exchanges. It is singular or remarkable in several particulars. First, on account of the *assumed* dignity, importance, and representative character of those whose names are signed to it. For they not only "*claim to be* better acquainted with the working of the Medical Department of the Army" than any one else, but, also, to be the representatives of "some millions of constituents," together with the "*science* of the country and the *humanity* of its homes."

Second, on account of the assumption that all the improvements and progress made in the Medical Department of the United States army are due "to the fact that for two years the Medical Department has been directed by Dr. W. A. Hammond, Surgeon-General." According to this body of men, representing "some millions of constituents," &c., all the hard-working, self-denying members of the medical staff of the United States army have been but simple clay in the hands of the "thoroughly scientific, highly endowed, large-minded, and energetic" Surgeon-General. And so destitute is the whole Staff of both brains and science, that not a man in it could be found qualified to fill the position of Surgeon-General, and consequently the removal of Dr. Hammond "would be as serious a blow at the lives, comfort, and efficiency of the army as the enemy itself could inflict." Alas! what if the Surgeon-General should die!

Third, it is most remarkable of all, on account of the demand for a court to try Dr. Hammond, after they, in their character as "*experts*," have fully pre-judged his case, by declaring that "his administration has *more* than justified all the high hopes and expectations of those who recommended him for the place."

Now, we are heartily tired and nauseated with such arrogant bombast and pretension as is put forth in the above document. We do not know what charges have been preferred against Dr. Hammond, as Surgeon-General; and we sincerely hope that they will be fairly and fully investigated, and for the credit of the profession to which he belongs, that they will be wholly disproved. But we certainly have no idea that all the science, the humanity, and the energy of the Medical Department of the Army is centered in him; or that his removal would endanger the dissolution of the Army. On the contrary, we believe the Medical Staff of the army contains a score of men, any one of whom are quite as competent to fill the office of Surgeon-General as is Dr. W. A. Hammond.

If Dr. Hammond is a man of such remarkable endowments, and peculiar fitness for the post of Surgeon-General, we would respectfully ask the above committee of "experts" why it is that after two years of his administration there is not yet an efficient ambulance system for attending properly to the wounded in battle; and why the thousands of sick and wounded in all departments of the army are still pensioners on the *charity* of the nation, instead of having all their legitimate wants supplied promptly through the regular and responsible officers of the Medical and Commissary Departments? Is it any credit to the Government, or proof of efficiency on the part of the Secretary of War and Surgeon-General, to have the army so managed that no less than $7,000,000 or $8,000,000 of gratuitous and charitable donations have been required from the people to keep the sick and wounded soldiers from unnecessary suffering? Finally, is it the proper business of a *Sanitary Commission* to dispense *charity*, or to investigate and expose those causes that injure the health of the army, and the most reliable means for avoiding them?

---

THE MEDICAL STAFF OF ENGLAND.—From the last census it appears that there are, in England and Wales, one surgeon or general practitioner to about 1,712 of the population, one physician to 5,552, and one dentist to 3,505.

---

### ON THE VALUE OF TINCTURE OF IODINE AS A TEST FOR DIABETIC URINE.

(*To the Editor of The Lancet.*)

SIR:—Having a case of diabetes mellitus under my care, and wishing to ascertain the value of the recently discovered test of Trousseau and Dumontpallier, I performed the following experiment:—

Equal quantities of healthy and diabetic urine were placed in two test-tubes (sugar having been ascertained to be absent from the former specimen). I then added to each fifteen drops of tincture of iodine. The effect was to tinge the healthy urine of a rose color, which it retained; while in the diabetic specimen all trace of color was promptly removed, showing the presence of glucose.

The test is very convenient, as well as very striking to the eye when compared with a healthy specimen. It is also very delicate, as I found by diluting the urine with water until I reached a specific gravity of less than 1001. In the latter experiment all color was removed, but it took a longer time.

The presence of rhubarb has appeared to me to interfere with Luton's test (bichromate of potash and sulphuric acid); but it produces no such effect upon the iodine test.

Should the value of this test be further confirmed, it will be found more ready and convenient than any of the means hitherto employed for the detection of sugar in the urine.

I am, Sir, your obedient servant,
H. D. Massy, Staff Assist.-Surg.

---

### LETTER FROM NEW YORK.

### THE NEW (OR OLD) ANÆSTHETIC.

*Editor Medical and Surgical Reporter:*

Sir:—Being informed that you take an interest in the question as to whom the honor belongs of having discovered and made practical anæsthesia, I ask leave to bring to your notice some facts within my own knowledge, which would seem to conclude the whole matter.

No one, I think, who has taken pains to examine the evidence on the subject, can doubt that the honor of the discovery belongs to the late Dr. Horace Wells, of Hartford, Connecticut. He made his discovery at one of my "exhibitions" of the "nitrous oxide gas," in the city of Hartford, on the evening of the 10th of December, 1844, and the next day he brought that discovery to the test of an experiment on himself. I, at his request, administered to him the gas, and Dr. Biggs, a dentist, extracted one of his large molar teeth without the slightest pain. The success of the operation surprised us all. This was, no doubt, the first successful trial of an adequate anæsthetic agent, and led to all that has since followed in this beneficent line of effort.

He attempted to establish the truth and value of his discovery in Boston, soon after, calling on Dr. Jackson, Dr. Morton and many Dentists of that city. These facts are established by an elaborate and unanimous report on the question "who discovered anæsthesia," made to the New York Legislature at the session of 1860, by the Medical Society of that State. This report contains an exhaustive consideration of the claims of Wells, Jackson and Morton, arriving at the conclusion that to Wells alone belongs the honor of the discovery. The facts are also established by many depositions, gathered and published in a volume on "anæsthesia," prepared by the Hon. Truman Smith. By these depositions Mr. Smith shows that Dr. Wells was in the constant and successful use of the nitrous oxide gas as an anæsthetic, in the city of Hartford, during nearly the whole of 1845. He occasionally used ether, but preferred the nitrous oxide for a brief operation, like the extraction of teeth. During this time the subject of anæsthesia had not obtained any hold on the public mind, except in the city of Hartford. The discoverer encountered almost insuperable incredulity. It could not be believed that *any* substance could be breathed so as to destroy pain in surgical operations! Surgeons and the medical profession treated the subject as chimerical. It is no wonder that Dr. Wells, (a dentist,) made very slow progress in the introduction of his new discovery.

In December, 1845, Dr. Wells went to Europe to bring out his new invention, during his absence Drs. Jackson and Morton commenced a series of experiments with ether, to determine what there was in this discovery; their experiments proved successful; their next step was to secure a patent, which they applied for in the month of *November*, 1846, nearly two years after Wells made and demonstrated his discovery. Before it was issued, Jackson assigned his interest in the patent to Morton, therefore the patent was issued in the name of Morton. This, be it remembered, was all done in the absence of Wells. On the return of Wells to this country a discussion commenced between him and Morton as to the origin of the discovery, during which Wells died, leaving a widow in indigent circumstances with no means to prosecute the matter, consequently Morton had the field clear to tell his own story. By the disinterested and unpaid labors of the Hon. Truman Smith the testimony was gathered which establishes beyond cavil the truth of the above statements.

At the time of the death of Wells, very few people, aside from eye-witnesses, had any faith in either nitrous oxide or ether as an anæsthetic. After this event the nitrous oxide was superceded by the use of ether, so far as any anæsthetic was used. Ether could be purchased at any drug store, while it required expensive apparatus and some chemical knowledge to make the nitrous oxide, so that for twenty years past the nitrous oxide has hardly been thought of as an anæsthetic, till I revived it in May last, in New Haven, Connecticut. I there announced that I believed the nitrous oxide was a safe and efficient anæsthetic for the extraction of teeth, and on account of its harmlessness, was far superior to ether or chloroform. I arranged with Dr. Smith to extract teeth by this new (or old) anæsthetic, and in the space of three weeks and two days we extracted *over eight thousand teeth*, and without any pain to the patients. I then came to New York and established the "Colton Dental Association," and have administered the gas, up to the present time, for the extraction of about eight thousand teeth. We find it works with far more certainty and uniformity than ether or chloroform, for we have never yet found a patient whom we could not put into a profound sleep. In no instance has any injurious effect attended the operation. We have given it to persons suffering from almost all sorts of disease, nervous debility and weakness, and heart disease, including many cases of far gone consumption, with no ill effects. In no instance have patients complained of pain. In a very few cases, where a large number of teeth have been extracted at a sitting, and several doses had to be given, a little blood would be swallowed and nausea would result. With these rare exceptions, not the slightest unpleasant effects have followed.

We have never had a patient who had previously taken ether or chloroform that did not warmly express his preference for nitrous oxide. Hundreds of dentists, who were, at first, skeptical about the nitrous oxide *on seeing its operation*, have given it their warmest commendation, and I do not now know of *one* in New York, who does not prefer it to ether or chloroform. The opinion on this subject, so far as I have been able to learn, is universal, and a large number send us their patients requiring the extraction of teeth.

I think these facts ought to satisfy the medical, also the profession and public, that for a brief surgical operation, the nitrous oxide gas is the best anæsthetic known. I have no doubt that if desired, I could keep the patient in an anæsthetic state for fifteen minutes or more. Instead of producing anæsthesia by depressing the vital forces, and diminishing the circulation of the blood in fact, approaching the point of death, the gas produces anæsthesia from the very opposite cause, from *over exhilaration*, and from an increased and more highly oxygenized condition of the blood. A small dose of nitrous oxide or ether or chloroform will exhilirate, a larger induces sleep. But ether and chloroform act directly on the nerves, and as a *stimulant.* A consequent reaction and depression follows. The nitrous oxide is not a stimulant, and does not act directly on the nerves at all. The gas is taken into the lungs and is rapidly absorbed by the blood, producing a highly oxygenized condition of that fluid. This highly purified blood acts as an *exhilarant* on the nerves. (There is a wide difference between an exhilarant and a stimulant.) The anæsthetia is produced by *over exhilaration.* There is no reaction or depression following its inhalation. When I say this, I but reiterate what all works on chemistry state. I speak also from an experience of twenty years with the gas.

I call nitrous oxide the new anæsthetic, because I have after a long interval, revived the use of it. But it is in fact the old anæsthetic of Horace Wells, and I rejoice that Providence has permitted me to live and demonstrate the truth and validity of his great discovery. Surgeons, physicians, and dentists from any part of the country who may desire to verify the truth of the above representations, are invited to call and witness the operations at the rooms of the Colton Dental Association, 22 Bond street, New York.

Respectfully, G. Q. Colton.

---

Chicago Medical Journal.—We see by the January number of this monthly, that it has again changed hands.

Professors Brainard and Allen, its former editors and proprietors, have transferred it to Professors Miller and Ingalls, of the same faculty. We congratulate its readers on this change, for if its future editorial articles should be less facetious and spicy, they will doubtless be more truthful. We welcome the new editors as co-laborers in the field of medical journalism.

---

### GLYCERINE.

This may appear at first sight an almost exhausted subject; but the uses of glycerine in medicine and surgery are so numerous that there are still many points worthy of notice.

The therapeutic uses of glycerine may be said to have begun with its employment in cases of deafness by Mr. Thos. Wakley, in 1849. Mr. Startin had, we believe, used it to some extent previously in skin diseases; but its value in aural surgery led directly to the recognition of its peculiar properties, and their adaptation to many practical requirements. It happened, fortunately, that shortly after the publication of Mr. Wakley's work a method was discovered of preparing glycerine perfectly pure. The whole range of pharmacy supplies no more striking illustration of the supreme importance of absolute purity in our remedial agents. So long as we were unable to obtain a pure glycerine, it was impossible to ascertain its real merits as a remedy. This desideratum was supplied by the industry and scientific skill of Mr. G. F. Wilson, the well known manager of Price's Candle Company. That manufactory, in his hands, has in a most remarkable manner demonstrated that scientific discoveries which at first sight appear quite useless or remote from any practical application may become of the greatest value in the common affairs of life. When chemists read with admiration the researches of Chevreul on fatty bodies, about forty years ago, they could scarcely have dreamed of the results to commerce and medicine which would flow from them. We doubt, indeed, whether even yet physiologists have duly appreciated them and their bearings on the function of fat in the living economy. When Mr. Wilson directed his attention to glycerine as a product of the operations on fats in candle making, it was a useless waste material, and hundreds of tons were thrown away annually. At that time we could only employ an impure fluid scarcely affording a glimpse of its real properties. The early attempts to employ glycerine gave rise to contradictory and conflicting opinions. This arose simply from the various matters attaching to it, from its mode of preparation, and the sources whence it was derived. It had a disagreeable odor from the presence of volatile fatty acids, and often contained lime, lead, and other metals, or metallic salts, sulphuric acid, hydrochloric acid, and alkaline or earthy salts. No wonder that it was found to irritate inflamed surfaces and to aggravate painful states—effects directly opposite to those of pure glycerine. As it is said there is still impure and adulterated glycerine in the shops, it is of primary importance to recognise the pure.

Glycerine should be absolutely without smell or color, with a saccharine taste, and of the consistence of syrup. Its chemical formula is $C^6 H^7 O^5 , H O$. With a specific gravity, at 60° F., of 1.24, it contains 94 per cent. of anhydrous glycerine. It can be concentrated to 1.26, when it contains 98 per cent. It is soluble in all proportions in water and alcohol, but insoluble in ether. It should show no reaction with litmus paper, and yield no precipitate with any reagent. Such is the glycerine prepared by Mr. Wilson, and known as Price's. Its source is palm oil, from which it is prepared by distillation in steam at a temperature of from 550° to 600° F. It is a curious fact, that although thus separated from its natural combination with fixed fatty acids, it will not again combine with them. Attempts to form preparations by combining glycerine with solid fats or oils are false in principle. Its combination with starch in various proportions, as described by Dr. Tilt, is the form in which it is best adapted for use; and it appears probable that this will in a great measure supersede all other ointments, whilst its solvent power renders it a most suitable vehicle for substances designed to be used with some friction on the surface as liniments and embrocations.

Unlike oils and fat, it does not absorb oxygen, and therefore never becomes rancid, or decomposes substances dissolved in it. It is probably mainly by virtue of this property that it acts as an antiseptic. Applied alone, it soothes the irritation of most skin diseases, and allays the pain of inflamed parts. With the exception of the formula for the use of starch to render it semi-solid, as given by Dr. Tilt, we see no advantage in copying the various formulæ which have been published. Every one can describe the remedy he selects according to its known properties, if the quantity soluble in the glycerine is known. Hence, we conceive the following tables will be found very useful:—

A. *Inorganic substances.*

100 parts of pure glycerine dissolve—

| | | | |
|---|---|---|---|
| Sulphur | 0-1 | Cyanide of mercury | 27-0 |
| Phosphorus | 0-2 | Arsenic acid | 20-0 |
| Iodine | 1-0 | Arsenious acid | 20-0 |
| Bromine | (all proportions) | Boracic acid | 10-0 |
| Monosulphuret of potassium | | Chlorate of potash | 3-5 |
| | (all proportions) | Arseniate of " | 50-0 |
| " of sodium " | | " of soda | 50-0 |
| " of lime " | | Carbonate of " | 98-0 |
| Persulphuret of potassium | 25-0 | Bicarbonate of " | 8-0 |
| Iodide of sulphur | 1-6 | Borate | 60-0 |
| " potassium | 40-0 | Carbonate of ammonia | 20-0 |
| " zinc | 40-0 | Hydrochlorate of " | 20-0 |
| Biniodide of mercury | 0-3 | Alum | 40-0 |
| Bromide of potassium | 25-0 | Sulphate of iron | 25-0 |
| Iodide of iron | (all proportions) | " zinc | 35-0 |
| Chloride of sodium | 20-0 | " copper | 30-0 |
| " barium | 10-0 | Nitrate of silver | |
| " zinc | 50-0 | | (all proportions) |
| " iron | | Tannic acid | 50-0 |
| | (all proportions) | Oxalic acid | 15-0 |
| Bichloride of mercury | 7-5 | Benzonic acid | 10-0 |
| Cyanide of potassium | 32-0 | | |

With the sulphuric, nitric, phosphoric, hydrochloric, acetic, citric, and tartaric acids, glycerine unites in all proportions; and the same with the caustic alkalies and some salts, as the hypochlorites of soda and potass. Most of the metallic salts soluble in water are to about the same degree soluble in glycerine; some, however, are decomposed, as the bichromate and permanganate of potass.

B. *Organic substances, alkaloids, &c.*

100 parts of glycerine dissolve—

| | | | |
|---|---|---|---|
| Morphia | 0-45 | Quinine | 0-5 |
| Muriate of morphia | 20-0 | Sulphate of quinine | 2-75 |
| Atropine | 3-0 | Cinchonine | 1-5 |
| Sulphate of atropine | 33-0 | Veratrine | 1-0 |
| Strychnine | 0-25 | Brucine | 2-25 |
| Sulphate of strychnine | 22-5 | Codeine | (all proportions) |
| Nitrate of strychnine | 4-0 | | |

Watery extractions of vegetables are very soluble in glycerine; whilst gums, resins, essential oils, ethereal extracts, camphor, balsams, fatty acids, are either wholly insoluble or sparingly soluble.

The internal uses of glycerine, either alone or in combination, or as a vehicle for active substances, await further investigation.

Notwithstanding that we are told the quantity of glycerine used in the hospitals of Paris increased from 300 pounds in 1851 to 3000 in 1861, we fear the demand for glycerine for therapeutic purposes will fall very far short of the amount produced as a residue in candle making, when this is reckoned at hundreds of tons per annum. That the demand for it will greatly increase as its value becomes recognised there is no doubt. If the price could be considerably reduced, the consumption would be much larger.

---

HÆMOSTASIS.—At the last meeting of the British Association in Newcastle, a paper was read by Dr. Junod, so well known as the inventor and improver of an apparatus for the purpose of producing a vacuum over certain portions of the body. By this instrument, as is now well known, the power is afforded of withdrawing rapidly into a given part of the body from the general circulation from six to ten pounds of blood, and to maintain this important displacement as long as the case requires. Thus the most important effects of venesection may be produced without the debility consequent upon absolute abstraction of a large quanity of blood.

---

TEST FOR SULPHUR.—A solution of molybdate of ammonia in diluted hydrochloric acid, is stated by M. Schlossberger to be a most delicate test for the presence of sulphur.

